# The Gut Microbiota and Autism Spectrum Disorder: Current Research and Therapeutic Insights

**DOI:** 10.3390/bs16040559

**Published:** 2026-04-08

**Authors:** Miao Zheng, Xueying Wei, Rui Chen, Chongying Wang, Lingbiao Xin

**Affiliations:** 1 State Key Laboratory of Experimental Hematology, Tianjin Medical University, Tianjin 300071, China; zhengmiao@tmu.edu.cn (M.Z.); cr13132232366@tmu.edu.cn (R.C.); 2 Key Laboratory of Cellular and Molecular Immunology in Tianjin, Tianjin Medical University, Tianjin 300071, China; 3 Key Laboratory of Immune Microenvironment and Disease (Ministry of Education), Tianjin Medical University, Tianjin 300071, China; 4 The Province and Ministry Co-Sponsored Collaborative Innovation Center for Medical Epigenetics, School of Basic Medical Science, Tianjin Medical University, Tianjin 300071, China; 5 Department of Biochemistry and Molecular Biology, School of Basic Medical Science, Tianjin Medical University, Tianjin 300071, China; 6 Department of Immunology, School of Basic Medical Science, Tianjin Medical University, Tianjin 300071, China; 7 Autism Research Center, School of Sociology, Nankai University, Tianjin 300071, China; xueying.wei@mail.utoronto.ca

**Keywords:** Autism Spectrum Disorder, gut microbiota, probiotics, prebiotics, fecal microbiota transplantation, therapeutic directions

## Abstract

Autism Spectrum Disorder (ASD) is a collective term for neurodevelopmental disorders with core features of social communication impairment, restricted and repetitive behaviors, and narrow interests. These include classic autism, Asperger’s syndrome, and pervasive developmental disorder not otherwise specified. ASD is currently managed with behavioral interventions, rehabilitation training, and family support, but there is no curative medication. Recent studies suggest that some patients with ASD may experience gastrointestinal symptoms. Perhaps this is associated with the disturbances of gut microbiota. Increasing evidence has demonstrated that the composition of gut microbiota in ASD individuals is different from that in normal population and may be associated with neurodevelopmental processes via the gut–brain axis. This article reviews the evidence for the association between gut microbiota and ASD, describes the characteristics of microbial changes, and analyzes the mechanism by which changes in the composition of the microbiota affect the occurrence and development of ASD. Finally, we review recent advances in microbiota-targeted therapeutic strategies, including probiotics, prebiotics, and fecal microbiota transplantation, which provide new approaches to alleviate and improve autism-related symptoms and point out the future research direction.

## 1. Introduction

Autism Spectrum Disorder (ASD) is a neurodevelopmental condition with onset in infancy, typically emerging before the age of 3 and persisting throughout life. Its core symptoms include impaired social communication, restricted and repetitive behaviors, and narrow interests. Additionally, ASD is frequently accompanied by co-occurring neuropsychiatric conditions such as intellectual disability, anxiety, and depression (Burrows et al., 2021; Mutluer et al., 2022; Ozyurt & Dinsever Elikucuk, 2017). Over the past two decades, reported ASD prevalence has risen globally, reflecting improved screening tools, expanded diagnostic criteria (particularly the 2013 DSM-5 integration of Asperger syndrome and PDD-NOS), and heightened public awareness (American Psychiatric Association, 2013). According to the CDC Autism and Developmental Disabilities Monitoring (ADDM) Network (2022 surveillance data, released April 2025), approximately 1 in 31 US children aged 8 years (3.2%) were identified with ASD through medical and educational record review across 16 communities—substantially higher than the 1 in 54 rate reported in 2016 (Shaw et al., 2025; Z. Wang et al., 2025). In contrast, global modeling estimates from 2021 suggest approximately 61.8 million people worldwide were affected (788 per 100,000, or 1 in 127), though these figures rely on heterogeneous methodological approaches differing from the US record-based surveillance system (Santomauro et al., 2025). In China, a 2013 national survey using multi-stage cluster sampling found 0.70% prevalence (1 in 143) in children aged 6–12 years, with 68.8% having co-occurring neuropsychiatric disorders (Zhou et al., 2020). These divergent methodological approaches highlight that prevalence figures are inherently context-dependent, shaped by local diagnostic practices, resource availability, and surveillance capacities. However, all these data indicate that ASD has now become a major global public health challenge, imposing substantial medical and social burdens.

**Figure 1:**
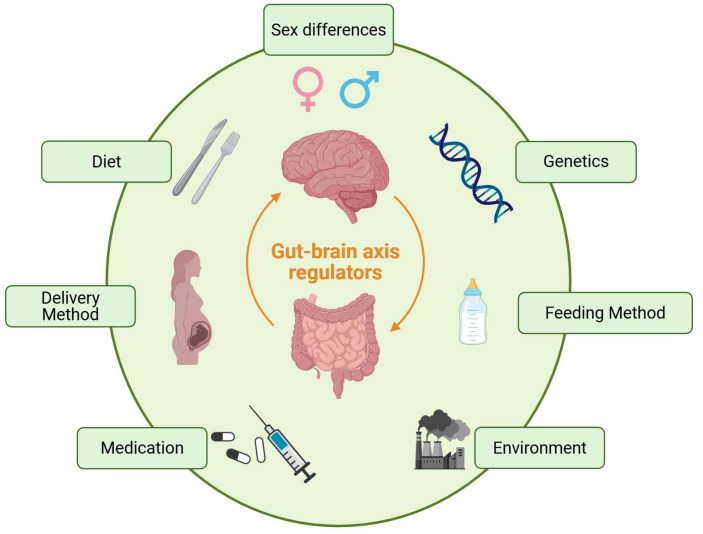
Schematic of Risk Factors in ASD (main including genetics, diet, sex, delivery method, medication, feeding method, and environmental factors).

The etiology and pathogenesis of ASD are not yet very clear. It is believed that it is a complex process that is influenced by multiple factors, including genetics, diet, sex, prenatal and perinatal conditions, and environmental factors (Figure 1) (Alharthi et al., 2022). These factors may induce the occurrence and development of ASD by disrupting the biological process through the participation of synaptic function, the oxytocin system, immune homeostasis and mitochondrial function (Karimi et al., 2017; Liu et al., 2023; Rylaarsdam & Guemez-Gamboa, 2019; C. Wang et al., 2017). Current treatments for ASD primarily include behavioral therapy, pharmacological interventions, and special education. Behavioral therapy is the mainstay approach, with common methods such as Applied Behavior Analysis (ABA), Cognitive Behavioral Therapy (CBT), and social skills training. These therapies aim to enhance social adaptability and quality of life, as well as modify maladaptive cognitive and behavioral patterns (Qin et al., 2024; Reichow et al., 2012; Roane et al., 2016). Medications may be used to alleviate certain symptoms (e.g., antidepressants or antipsychotics). Special education helps improve communication, self-care, and social adaptation skills through systematic training, thereby enhancing the quality of life for affected children (Hellings, 2023; Sharma et al., 2018).

However, the effectiveness of the above treatments is limited and needs to be maintained for a long time, which brings a huge burden of health care and education resources and brings huge economic and psychological burdens to families. In addition, the high degree of individual variability of ASD and the differences in treatment needs also make the diagnosis and treatment of ASD more complicated, which leads to increasing complexity and costs. Therefore, it is still a great challenge to find more efficient and convenient treatment methods and further understand the pathogenesis and mechanism of ASD.

Therefore, this article presents a narrative review synthesizing the current evidence on the association between the gut microbiota and autism spectrum disorder (ASD), with a focus on microbial dysbiosis characteristics, underlying mechanisms along the gut–brain axis, and emerging microbiota-targeted therapeutic strategies. A structured literature search was conducted in the PubMed and Web of Science databases, covering the period from January 2010 to March 2026, and was restricted to English-language publications. The search strategy employed the following Boolean combination: (“autism spectrum disorder” [Title/Abstract] OR “ASD” [Title/Abstract] OR “autism” [Title/Abstract]) AND (“gut microbiota” [Title/Abstract] OR “gut microbiome” [Title/Abstract] OR “intestinal microbiota” [Title/Abstract] OR “probiotics” [Title/Abstract] OR “prebiotics” [Title/Abstract] OR “fecal microbiota transplantation” [Title/Abstract] OR “FMT” [Title/Abstract]). No additional filters were applied to maximise sensitivity. Retrieved records were imported into reference management software for duplicate removal and subsequent screening.

Inclusion criteria were: (1) peer-reviewed original research articles, systematic reviews, and meta-analyses; (2) studies investigating the association between gut microbiota and ASD in human subjects or animal models; and (3) studies published in English. Exclusion criteria were: (1) case reports, conference abstracts, and editorials; (2) studies lacking a clear ASD diagnosis or microbiota assessment; and (3) duplicate publications. Priority was given to human studies over animal models, and to recent high-quality original research articles, systematic reviews, and meta-analyses where available. Additionally, the reference lists of included articles and relevant review papers were manually searched to identify further studies.

## 2. The Link Between Gut Microbiota and Autism

The gut microbiota is important symbiotic ecosystem in the human body participating in the regulation of digestion and metabolism, immunity, barrier and neuroendocrine, etc., and is essential for the maintenance of body homeostasis (Fan & Pedersen, 2021). According to reports, some patients with ASD exhibit gastrointestinal symptoms such as constipation, diarrhea, and abdominal pain (Jolanta Wasilewska & Klukowski, 2015). This phenomenon has prompted researchers to explore the potential role of gut microbiota in the pathogenesis of ASD. Recent research has gradually uncovered the “microbiota–gut–brain axis” as a bidirectional communication pathway (Góralczyk-Bińkowska et al., 2022; Gulas et al., 2018). This provides a new theoretical foundation and therapeutic approach for intervening in ASD symptoms by modulating the gut microbiota.

### 2.1. Evidence Linking Gut Microbiota and ASD

In a 2019 animal study, researchers proposed a potential association between intestinal microbiota dysbiosis and ASD in animal models. By transplanting the fecal microbiota of autism spectrum disorder (ASD) patients into germ-free mice, the researchers successfully induced core ASD behavioral phenotypes in the recipient mice, such as social deficits and increased repetitive behaviors. Metabolomic analysis of colonic contents from 20 oASD mice and 15 oTD mice revealed that gut microbial metabolites were significantly altered, with oASD mice exhibiting ~50% less taurine compared to oTD mice. Additionally, 5-aminovaleric acid (5AV) was significantly decreased, while 3-aminoisobutyric acid (3AIBA) was significantly elevated in oASD mice. These findings indicate that the microbiota and microbiota-derived metabolites may be involved in the regulation of ASD-related behaviors (Sharon et al., 2019). While animal models offer valuable mechanistic insights into causality, they do not fully recapitulate the complexity of human pathophysiology. Complementing these experimental findings, a large-scale clinical study involving 1627 children (aged 1–13 years, 24.4% female) with or without ASD provided pivotal translational evidence at the population level, confirming the potential association between ASD and human gut microbiota dysbiosis. Through metagenomic sequencing of faecal samples, the study revealed that children with ASD exhibited significantly decreased gut alpha diversity, along with multi-dimensional alterations involving 14 archaea, 51 bacteria, 7 fungi, 18 viruses, 27 microbial genes, and 12 metabolic pathways (Su et al., 2024).

In summary, animal studies have provided evidence for the causal role of gut microbiota in ASD-like behaviors, while clinical studies have confirmed the potential association between gut dysbiosis and ASD in human populations.

### 2.2. Alterations in Gut Microbiota of ASD Patients

Although the specific changes in the gut microbiota of individuals with ASD are reported to be different in various studies [which may be related to factors such as age, sex and diet], microbial dysbiosis has been consistently reported in ASD patients (Alharthi et al., 2022).

Gut microbiota dysbiosis in ASD patients is mainly manifested by the imbalance of core bacterial phyla. As the most dominant gut microbiota, the ratio of *Firmicutes*/*Bacteroidetes* (*F*/*B* ratio) is closely related to diseases such as obesity and inflammation (Stojanov et al., 2020). Most of the studies reported an increased *F*/*B* ratio in the gut of ASD patients, which was usually characterized by an increase in *Firmicutes* or a decrease in *Bacteroidetes*, although a few studies reported the reverse trend. This may be attributed to factors such as sex, age, and diet (Alamoudi et al., 2022; Dan et al., 2020; X. Kong et al., 2019; Niu et al., 2019; Strati et al., 2017).

Additionally, in the gut microbiota of individuals with ASD, the functional category abundance of multiple beneficial bacteria continues to decline. Numerous studies have indicated that these alterations may be closely associated with the pathophysiological mechanisms of ASD. First, carbohydrate-fermenting bacteria involved in short-chain fatty acid production, including *Prevotella*, *Coprococcus*, and *unclassified Veillonellaceae*, show decreased abundance, which may affect the signaling of gut-derived metabolites to the central nervous system and thereby correlate with core symptoms of ASD (Kang et al., 2013; Kovatcheva-Datchary et al., 2015). Second, immunomodulatory bacteria such as *Bifidobacterium* species and *Akkermansia muciniphila* are significantly reduced, a change that may compromise gut barrier integrity and immune homeostasis, thereby contributing to neurodevelopmental abnormalities in ASD through immune pathways (Gavzy et al., 2023; Tomova et al., 2015; L. Wang et al., 2011). Third, potentially probiotic species, including *Lactobacillus reuteri*, *Faecalibacterium*, *Lactobacillus johnsonii*, *Bacteroides uniformis*, and *Bifidobacterium pseudolongum*, also exhibit reduced abundance (Buffington et al., 2016; Zhong et al., 2026). Previous studies have indicated that the reduction in these bacterial groups is associated with improvements in social behavioral deficits in animal models of ASD. Additionally, the abundance of other commensal genera such as *Streptococcus* and *Dialister* is decreased (Tao et al., 2025), and changes in these microbial populations have also been suggested to correlate with the clinical manifestations of ASD.

On the other hand, a trend of increasing abundance of some harmful microbes in gut microbiota is observed in individuals with ASD. The over-proliferation of *Escherichia* may consume GABA and result in the metabolic imbalance of GABA and behavioral symptoms (D. Wang et al., 2025). The increase in *Desulfovibrio*, which can produce hydrogen sulfide in a metabolic way, will aggravate intestinal pathologies. The abundance of *Clostridium cluster XVIII*, *Clostridioides difficile*, *Actinobacteria*, *Sutterella* and *Betaproteobacteria class* is also significantly increased (Morton et al., 2023; Socała et al., 2021; Zhou et al., 2024; Mi et al., 2026). Furthermore, dysbiosis is also presented in the fungal community. The abundance of *Candida albicans* is increased in the gut of children with ASD, and the ammonia and other toxins produced by *C. albicans* may induce behavioral abnormalities (Iovene et al., 2017).

In summary, gut microbiota dysbiosis in ASD shows three patterns: (1) The *Firmicutes/Bacteroidetes* ratio shows directional variability, likely reflecting age and dietary differences; (2) Beneficial bacteria is consistently reduced, associated with compromised gut barrier function and immune impairment; (3) Potentially harmful microbes are significantly expanded, linked to metabolic disruption and intestinal pathology. These alterations may contribute to the pathogenesis and progression of ASD through metabolic, immune, and neural mechanisms (Table 1).

## 3. Underlying Mechanisms of Gut Microbiota in Brain Development

**Figure 2:**
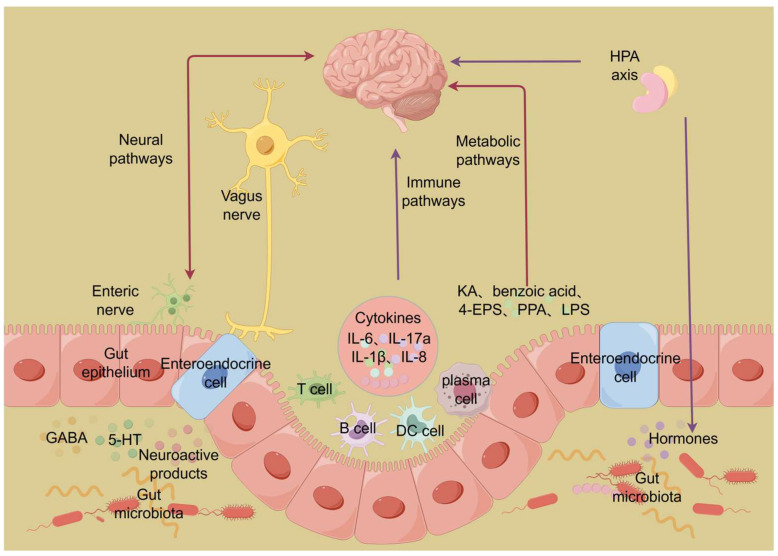
Gut–Microbiota–Brain Axis Schematic. The bidirectional communication between the gut microbiota and the brain involves several routes, including the immune, metabolic, and neuroendocrine routes. The results of the above mechanistic studies provide a theoretical basis for the use of microbiota for ASD.

The gut microbiota may play a role in the pathogenesis of ASD through multi-pathway interactions along the gut–brain axis, primarily influencing neurodevelopment and function via immune, metabolic, neural, and endocrine pathways (Figure 2) (Park et al., 2025a).

### 3.1. Immune Pathway

Immune pathways are integral to the bidirectional communication of the microbiota–gut–brain axis. The gut microbiota may regulate the inflammatory state of the brain by modulating the immune system. For example, it has been demonstrated that certain gut bacteria can modulate maternal Th17 immune response and induce the secretion of IL-17a, which modulates the development and behavior of offspring, resulting in social abnormalities and repetitive stereotyped behavior (Powell, 2017). Furthermore, when the gut microbiota is dysregulated, the integrity of the intestinal barrier will be weakened, and microbial products, such as lipopolysaccharide (LPS), can enter systemic circulation. This causes a whole-body inflammatory response and upregulation of pro-inflammatory cytokines (IL-6, IL-17a, TNF, IFN, IL-1β, IL-8). Critically, these cytokines can enter the brain via the blood–brain barrier and activate microglia, which may lead to sustained microglial activation and neuroinflammation, which in turn disrupts synaptic pruning and contributes to excitatory/inhibitory imbalance—key features of aberrant neurodevelopment in ASD (Hsiao et al., 2013; Kim et al., 2018; Lukens & Eyo, 2022). Moreover, other metabolites secreted by the gut microbiota, such as short-chain fatty acids, can also modulate the maturation of the host immune system and the balance of pro-inflammatory and anti-inflammatory responses. The imbalance in these metabolites may also contribute to neurodevelopmental abnormalities (Park et al., 2025b). Recent evidence further highlights that gut dysbiosis-induced neuroinflammation involves disruptions in immune signaling pathways such as NLRP3 inflammasome and NF-κB signaling, which are implicated in ASD pathogenesis (Zarimeidani et al., 2024). Recent 2026 studies have further elucidated the molecular mechanisms linking specific microbial taxa to immune dysregulation in ASD. Large-scale Mendelian randomization analyses have established that depletion of *Turicibacter*, *Streptococcus*, and *Lachnospiraceae NK4A136* is causally associated with ASD susceptibility, mediated through altered immune responses and intestinal barrier dysfunction (Wu et al., 2026). Collectively, immune-mediated microglial activation and subsequent synaptic dysfunction may represent a crucial link between gut microbiota dysregulation and ASD-specific neural circuit abnormalities.

### 3.2. Metabolic Pathway

Additionally, gut microbiota can release some metabolites that regulate brain development via metabolic processes. For example, microbial-derived 4-ethylphenyl sulfate (4-EPS) is absorbed into systemic circulation, crosses the blood–brain barrier, and affects the maturation and function of oligodendrocytes, which may lead to abnormal myelination and altered neural activity and functional connectivity (e.g., in emotion-regulation-related brain networks)—a mechanism that has been linked to social behavior deficits in ASD. Clinically, elevated serum 4-EPS levels have been associated with increased severity of restrictive and repetitive behaviors in ASD patients, suggesting its potential as a peripheral biomarker for ASD symptom severity (Diaz Heijtz et al., 2022; Foster, 2022). In addition, other studies have revealed that Lactobacillus can modulate GABA metabolism. GABA is the major inhibitory neurotransmitter in the CNS; its dysregulation, often reflected as an imbalance between excitation and inhibition (E/I imbalance) at the synaptic level, may be involved in the pathogenesis of anxiety and emotional regulation disorders commonly comorbid with ASD (Bravo et al., 2011; Cryan & Kaupmann, 2005). When the concentration of microbial metabolite propionic acid (PPA) is excessively high, it displays neurotoxic properties: it not only induces cerebral oxidative stress, mitochondrial dysfunction, and microglial activation—which may lead to motor impairments, anxiety, cognitive deficits, and gastrointestinal symptoms—but also heavily stimulates the proliferation and survival of human neural stem cells and enhances the differentiation of glial cells. These effects collectively disrupt neural circuit formation and sustain neuroinflammation, suggesting a potential link between metabolic dysregulation and ASD-associated synaptic dysfunction and altered neurodevelopment (Abdelli et al., 2019; Thomas et al., 2012). Benzoic acid is an aromatic compound produced by different strains of Lactobacillus; its metabolic pattern differs in ASD. It has been reported that the abundance of Lactobacillus in ASD children is reduced, accompanied by decreased benzoic acid production, which may aggravate ASD behavior (T. Li et al., 2025). Moreover, the levels of certain tryptophan-derived microbial metabolites—especially the neuroprotective kynurenate (KA)—are significantly decreased in the feces of ASD children. The metabolic disturbance induced by ASD may affect ASD symptomatology via the alteration of neural activity in some brain regions, e.g., the insula and cingulate cortex (Aziz-Zadeh et al., 2025). Clinically, the kynurenine pathway ratio (KA/QA) can serve as a potential diagnostic biomarker. Together, these metabolic pathways have been implicated in key ASD-related mechanisms, including neurotransmitter imbalance, synaptic dysfunction, and microglia-mediated neuroinflammation.

### 3.3. Neural Pathways and Neuroendocrine Pathways

In terms of neural pathways, gut microbes can remotely influence brain function by transmitting signals through the vagus nerve (Sgritta et al., 2019). For instance, neuroactive metabolites produced by the gut microbiota, such as amino acids and neurotransmitter precursors (e.g., serotonin (5-HT), dopamine, and glutamate), can travel along the vagus nerve or enter the bloodstream to reach the brain, thereby regulating the homeostasis and function of the nervous system (Caspani & Swann, 2019; Y. Chen et al., 2021). Notably, in ASD, dysregulation of neurotransmitter systems such as serotonin and glutamate has been shown to be closely associated with synaptic dysfunction and aberrant neural connectivity, suggesting a potential link between the gut microbiota and ASD-related neurochemical imbalances. In terms of neuroendocrine pathways, gut microbes can influence systemic hormone levels by modulating the function of intestinal hormones, such as through their effects on enteroendocrine cells (EECs). Enteroendocrine cells secrete serotonin (5-HT), glucagon-like peptide-1 (GLP-1), and peptide YY (PYY), which modulate brain function either by entering the bloodstream or by directly activating the vagus nerve (Bosi et al., 2020). Meanwhile, the gut microbiota can also regulate the function of the hypothalamic–pituitary–adrenal (HPA) axis, which plays a critical role in stress responses and neurodevelopment. Overactivation of the HPA axis can lead to abnormally elevated cortisol levels, which in turn adversely affects neurodevelopment and social behavior (Chidambaram et al., 2022).

The microbiota–gut–brain axis constitutes a bidirectional loop, wherein dysfunction in any component may contribute to disease. Current evidence indicates that the synergistic dysregulation of immune, metabolic, neural, and neuroendocrine mechanisms collectively contributes to the pathological features of ASD, including synaptic dysfunction, microglial activation, neuroinflammation, and neurotransmitter imbalance.

## 4. Microbiota-Targeted Intervention Strategies

**Figure 3:**
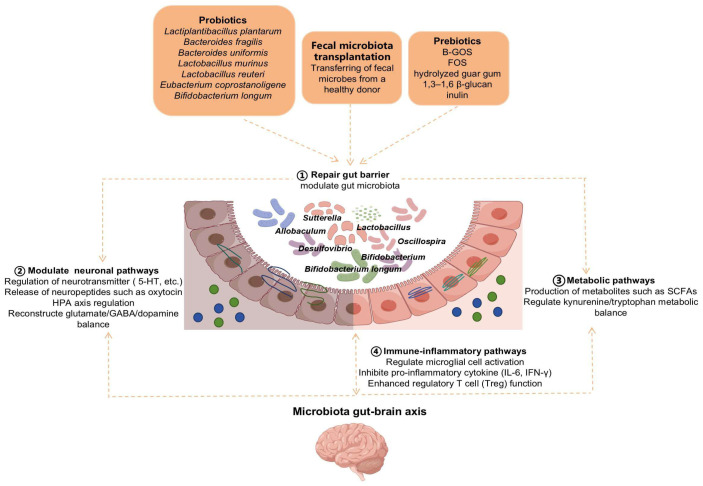
Microbiota-Targeted Therapeutic Strategies (by repairing gut barrier, thereby regulating neural, immune and metabolic pathways). 1. Probiotics (*Lactiplantibacillus plantarum*, *Bacteroides fragilis*, *Bacteroides uniformis*, *Lactobacillus murinus*, *Lactobacillus murinus*, *Lactobacillus reuteri*, *Eubacterium coprostanoligene*, *Bifidobacterium longum*). 2. Prebiotics (B-GOS, FOS, hydrolyzed guar gum, 1,3–1,6 β-glucan, inulin). 3. FMT (Transferring of fecal microbes from a healthy donor). The above mechanistic studies provide a theoretical basis for the use of microbiota for ASD.

The aforementioned mechanistic research provides a theoretical foundation for microbiota-based interventions in ASD. Current approaches, including probiotics, prebiotics, and fecal microbiota transplantation (FMT), have demonstrated potential therapeutic value They primarily exert their effects by remodeling gut microbiota structure, modulating metabolites and immune responses, alleviating neuroinflammation, and restoring neurotransmitter balance, thereby improving core behavioral and gastrointestinal symptoms in ASD (Figure 3) (Wadan et al., 2025).

### 4.1. Probiotics Therapy

Probiotics have demonstrated potential application value in the prevention and treatment of various diseases. Their primary functions include regulating gut microbiota balance, enhancing intestinal barrier function, modulating the immune system, and improving digestive function. In recent years, many studies have reported that certain probiotics may exhibit therapeutic effects on ASD.

In terms of basic research, *Lactiplantibacillus plantarum* has exhibited considerable therapeutic effects. It has been reported that after a one-month intervention (from weaning at 1 month of age to 2 months of age), *Lactiplantibacillus plantarum PS128* (*PS128*) can greatly modulate the composition of gut microbiota in ASD mice (as assessed by 16S rRNA sequencing, especially the increase in the population of *Bifidobacterium*), recover the level of oxytocin in the hypothalamic paraventricular nucleus, and increase the activity of related protein kinases (Erk, CaMKII and PKA) in the hippocampus. The activation of these kinases can improve the morphology of dendrites and facilitate neuronal connection (C.-M. Chen et al., 2024). Another study revealed that after a four-week intervention (from adulthood, via food at a dose of 2 × 10^9^ CFU/g), *Lactiplantibacillus plantarum N-1 (LPN-1)* can remodel the gut microecology via the “gut–brain axis,” as assessed by 16S rRNA sequencing. This intervention not only significantly increases the abundance of beneficial bacteria such as *Allobaculum* and *Oscillospira* in the gut of ASD mice but also significantly reduces the abundance of harmful bacteria like *Sutterella* and *Desulfovibrio*. Additionally, probiotics *Bifidobacterium* and *Akkermansia* changed from “absent” to “present” after LPN-1 intervention. This intervention significantly alleviates neuroinflammation and improves social deficits and anxiety/depression-like behaviors in mice, providing a new candidate strain for the development of microbiota-targeted therapies (Qiu et al., 2023).

*Bacteroides fragilis* has also been demonstrated to possess therapeutic potential for ASD. In fundamental research, studies have found that after intervention with *Bacteroides fragilis* in ASD mice, the levels of 34% of the differential metabolites (such as 4-EPS) in the mice returned to normal, gut barrier integrity was improved, and the microbiota structure reverted to a non-ASD state. Concurrently, it significantly alleviated ASD-related behavioral abnormalities, including anxiety-like behaviors, in MIA mice (Gilbert et al., 2013). The above findings are based on fundamental research, which has been further validated in clinical studies. Following a 16-week intervention with *Bacteroides fragilis BF839* (10 g/bar with ≥10^6^ CFU/bar of viable bacteria, two bars/day, *n* = 60 children aged 2–10 years diagnosed with ASD), the abundances of *Bifidobacterium pseudocatenulatum*, *Bifidobacterium longum*, and *Bifidobacterium bifidum* in the gut of children with ASD significantly increased, accompanied by significant improvements in abnormal behaviors and gastrointestinal symptoms, with only two patients (6.67%) in the *BF839* group experiencing mild diarrhea (Lin et al., 2024).

*Bacteroides uniformis* and *Lactobacillus murinus* have also shown preliminary therapeutic potential in preclinical trials. *Bacteroides uniformis* was able to ameliorate ASD-like behaviors and neuro-excitation/inhibition balance following a 4-week intervention (gavage every 3 days starting from weaning) by modulating gut amino acid transport and microbiota composition (Yu et al., 2022). Intervention with Lactobacillus murinus for one month significantly improved social novelty deficits in Chd8ΔIEC mice, accompanied by an increased proportion of Drd2-positive neurons and the restoration of synaptic plasticity-related pathways (such as Gria2 and Snap25). It may remodel the interactive network between neurons and glial cells in the brain, potentially by modulating gut inflammation, metabolite secretion, or vagus nerve signaling, thereby significantly alleviating social novelty deficits in mice (Ji et al., 2025). Furthermore, intervention with *Lactobacillus murinus* also significantly improved the fecal microbiota composition in children with ASD, notably increasing the levels of *Bifidobacterium* and *Lactobacillus*, while the severity of both core ASD symptoms and gastrointestinal symptoms significantly decreased (Sivamaruthi et al., 2020).

*Lactobacillus reuteri* has shown good safety and efficacy in clinical trials. In a pilot double-blind randomized placebo-controlled trial involving 43 children with ASD (aged 5–13 years) who received 6-month (26-week) intervention, a combination product containing two strains of *L. reuteri* (*ATCC-PTA-6475* and *DSM-17938*) significantly improved social interaction deficits in children with ASD, with no observed negative impacts on the gut microbial community or immune system (Mazzone et al., 2024). Further research found that after five weeks of treatment, *Lactobacillus reuteri IMB015 (IMB015)* might alleviate ASD-related phenotypes following a 5-week intervention (daily oral gavage of 1 × 10^9^ CFU, from 3 weeks to 8 weeks of age) by reducing fecal glutamate levels and the glutamate/GABA ratio in mice, and decreasing 3-hydroxyglutaric acid levels, while increasing the number of Treg cells in the brain, reducing IFN-γ secretion by CD4^+^ and CD8^+^ T cells, and lowering IL-6 production by microglia (Park et al., 2025b).

Additionally, *Eubacterium coprostanoligene* has shown long-term improvement effects on related gastrointestinal (GI) and behavioral symptoms, suggesting potential benefits for alleviating ASD-related symptoms (N. Li et al., 2021). After an 8-week intervention, *Bifidobacterium longum CCFM1077* was able to correct the kynurenine pathway (KP) metabolism in the periphery (gut and blood) and the brain. Furthermore, it significantly reduced quinolinic acid (QUIN) levels in the brain, downregulated glutamate (Glu), increased GABA, normalized the Glu/GABA ratio, alleviated cerebellar microglial overactivation, and significantly ameliorated autism-like behaviors (repetitive/stereotyped behaviors, learning and memory deficits, and despair-like mood) (Q. Kong et al., 2022).

In conclusion, probiotics intervention in ASD demonstrates strain-specific effects: *Lactiplantibacillus plantarum (PS128*, *N-1)*, *Bacteroides fragilis BF839*, *Bacteroides uniformis*, *Lactobacillus murinus*, *Lactobacillus reuteri*, *Eubacterium coprostanoligene*, and *Bifidobacterium longum CCFM1077* show preliminary efficacy in improving social behavior, restoring gut barrier function, modulating neurotransmitter balance, and alleviating GI symptoms. However, well-powered randomized controlled trials are urgently needed to validate clinical efficacy and establish precision treatment protocols.

### 4.2. Prebiotic Therapy

Prebiotics are a class of functional food ingredients that can be selectively utilized by host microorganisms, positively influencing host health by promoting the growth and metabolic activities of beneficial bacteria. Recent studies indicate that prebiotics show potential application value in ASD.

For example, supplementation with galacto-oligosaccharides (B-GOSs) in an ASD gut model significantly increased the abundance of Bifidobacterium and altered the composition of specific bacterial groups such as *Clostridium*, *Roseburia*, and *Bacteroides*. This intervention also markedly modulated the short-chain fatty acid (SCFA) metabolic profile and elevated levels of ethanol and lactate. These microbial and metabolic changes were accompanied by improvements in autism-related behavioral symptoms (Grimaldi et al., 2017). Clinical studies further confirmed that following a 6-week intervention, B-GOS intervention not only enhanced gut microbiota diversity but also induced significant alterations in fecal and urinary metabolic profiles, including increased levels of creatine, dimethylglycine, carnitine, and citrate, along with decreased levels of amino acids and lactate, thereby significantly reducing antisocial behaviors in children with ASD (Grimaldi et al., 2018).

Moreover, in a study in which children with ASD were supplemented with hydrolyzed guar gum for 2 weeks, the authors also showed that the population of the beneficial bacteria increased, including *Bifidobacterium longum*, the serum pro-inflammatory cytokines decreased, constipation and behavioral irritability were significantly relieved, and ASD behaviors improved in general (Inoue et al., 2019; Ohashi et al., 2015). In addition, probiotics combined with fructo-oligosaccharides (FOSs) were able to reduce ASD phenotypes and modulate the serotonin level. Furthermore, 1,3–1,6 β-glucan was able to decrease the plasma α-synuclein levels and improve ASD phenotypes, as well as gut dysbiosis, during a 90-day period (Wu et al., 2025).

Furthermore, multiple researchers have reported that prebiotics capable of inducing short-chain fatty acid (SCFA) production can correct associated anxiety and depression-like behaviors. For example, inulin treatment was able to increase the levels of SCFAs and then improve the emotional behavioral abnormalities in mice withdrawn from ethanol exposure (K. Li et al., 2024). Moreover, *Lactiplantibacillus plantarum* was able to correct the epigenetic modification in the astrocytes of the offspring induced by maternal immune activation (MIA) by its metabolite sodium benzoate, which improved autism-like social behaviors through regulating gene expression by adjusting the epigenetic modification in a histone post-translational modification, histone benzoylation (T. Li et al., 2025). It provides theoretical basis for using *L. plantarum* as probiotics/prebiotics.

Another open-label, single-arm pilot study enrolled 30 children with ASD aged 4 to 11 years, who received a 12-week intervention with SCM06, a synbiotic formulation containing *Bifidobacterium bifidum*, *Bifidobacterium longum*, *Lactobacillus plantarum*, *Streptococcus thermophilus*, and a prebiotic. The results showed that SCM06 had a favorable safety profile; following the intervention, the children’s anxiety symptoms and sensory hyperresponsiveness significantly improved, and the prevalence of functional abdominal pain decreased from 26.7% to 10.0% (Wong et al., 2026).

In brief, prebiotics (B-GOS, hydrolyzed guar gum, FOS, β-glucan, inulin) promote beneficial bacteria and SCFA production, correlating with improved ASD behaviors and GI symptoms. The synbiotic SCM06 validates combined prebiotic–probiotic safety and efficacy. Rigorous clinical trials are needed to confirm benefits.

### 4.3. Fecal Microbiota Transplantation (FMT)

Fecal Microbiota Transplantation Abbreviation: FMT, also called intestinal microbiota transplantation, is a method in which the feces of healthy donors are subjected to rigorous processing and analysis to obtain functional microbial communities and their metabolites, which are then transferred into patients’ gastrointestinal tract to reconstruct the microecology of the gut, restore the intestinal mucosal barrier, and regulate the immune response and metabolic function (J.-W. Wang et al., 2019).

Recent studies have explored the application of FMT in the treatment of ASD. In one study, following a 3-week intervention (with 1-week antibiotic pretreatment and FMT administered every other day for 3 weeks), transplantation of fecal microbiota from healthy donors into autism model mice could effectively improve the core symptoms of autism, such as social interaction impairment and repetitive behavior. At the same time, FMT increased the diversity of the gut microbiota of autism model mice, and its composition approached that of healthy donors. In addition, FMT up-regulated the expression of genes involved in serotonergic and glutamatergic synaptic pathways in the brain of autism model mice (J. Wang et al., 2023). These results indicated that FMT was able to modulate brain function through the gut–brain axis by regulating the gut microbiota dysbiosis, which further affected the serum metabolites.

Another study applied the Microbiota Transfer Therapy (MTT) protocol, which included a 2-week course of oral antibiotics, bowel cleansing, and a high initial dose of FMT, followed by 7–8 weeks of daily low-dose maintenance treatments. The results showed that gut bacterial diversity in children with ASD significantly increased after MTT and remained higher than baseline levels even 8 weeks after treatment concluded. Additionally, the relative abundances of *Bifidobacterium*, *Prevotella*, and the *sulfate-reducing bacterium Desulfovibrio* were significantly increased post-treatment (Kang et al., 2017).

Multiple preliminary studies have shown that FMT may have potential in the management of ASD by modulating the gut microbiota and improving core behavioral and gastrointestinal symptoms in small-scale trials.

In summary, gut microbiota dysbiosis-mediated neuro-immune-metabolic disorders represent a key pathogenic mechanism of ASD. Probiotics restore microbial homeostasis in a strain-specific manner, repair intestinal barrier function, regulate neurotransmitter balance, and suppress neuroinflammation. Prebiotics and synbiotics enrich beneficial bacteria, elevate short-chain fatty acid levels, correct metabolic abnormalities, and mitigate immune activation. Fecal microbiota transplantation comprehensively reconstructs gut microecology and modulates cerebral synaptic pathways via the gut–brain axis, thereby improving core behavioral and gastrointestinal symptoms in ASD. These microbiota-targeted interventions coordinately regulate the neuro-immune-metabolic network and hold promising therapeutic potential; however, high-quality clinical studies are still required to establish precise intervention protocols.

## 5. Discussion

### 5.1. Clinical Significance of Microbiota-Related Findings

From a clinical perspective, the current findings on gut microbiota alterations in ASD have several practical implications. First, the consistent depletion of beneficial taxa (e.g., *Bifidobacterium*, *Akkermansia*, *Prevotella*) and enrichment of opportunistic pathogens (e.g., *Escherichia*, *Desulfovibrio*) provide a rationale for developing microbiota-based adjunctive biomarkers to aid in early screening or subtype classification, although they are not intended as standalone diagnostic tools. Second, the observed improvements in both gastrointestinal and core behavioral symptoms following probiotic, prebiotic, or fecal microbiota transplantation interventions suggest that modulating the gut–brain axis may serve as a complementary therapeutic strategy for a subset of ASD patients, particularly those with comorbid gastrointestinal disturbances. Third, the strain-specific and multi-pathway mechanisms (immune, metabolic, neural) imply that future clinical practice may move toward precision microbiome-targeted interventions rather than a one-size-fits-all approach. Nevertheless, these implications remain preliminary and require validation in well-powered clinical trials before translation into routine practice.

### 5.2. Methodological Challenges and Evidence Status

Despite the promising clinical implications, the field faces multiple methodological challenges that limit the current evidence base. First, phenotypic and biological heterogeneity. The ASD phenotype is highly heterogeneous, and differences in age, sex, severity, diet, antibiotic use, and comorbidities may significantly influence microbiome characteristics. Critically, most studies lack age- and sex-stratified analyses, even though emerging evidence indicates that both factors modulate ASD-associated microbial signatures. Second, technical variability across studies. There is considerable variability in microbiome sampling methods (e.g., stool vs. mucosa, different time points) and detection methods (e.g., 16S rRNA sequencing vs. metagenomics). 16S rRNA sequencing provides only genus-level resolution and cannot assess functional pathways, whereas metagenomics offers species-level and functional profiling (Table 2). This technological gap contributes to cross-study discrepancies and limits meta-analytic comparability. Third, limitations in study design. The current evidence is largely based on cross-sectional designs, making it difficult to establish causal relationships. The lack of longitudinal data and insufficient sample sizes limit temporal inference. Regarding the evidence base of therapeutic interventions, most studies have methodological limitations, including small sample sizes, the use of animal models or open-label designs, a lack of standardized clinical protocols, and insufficient long-term follow-up data. Although FMT has been described as promising in some studies, its evidence is derived primarily from small-scale pilot studies, and safety concerns (e.g., risk of infection transmission) have not yet been adequately assessed. Therefore, the clinical efficacy of these interventions should not be over-relied upon at present and requires further validation in rigorously designed randomized controlled trials. Fourth, quality variability in included studies. This review includes some small-sample pilot trials, whose main purpose is to explore intervention feasibility and estimate effect sizes, representing a relatively low level of evidence. It also includes a number of large-scale studies with a higher level of evidence, which can provide a more reliable basis for clinical decision-making. This heterogeneity in evidence quality must be fully considered when interpreting the results of this review.

### 5.3. Clinical Translation and Future Directions

Translating microbiome research into clinical practice for ASD requires overcoming major obstacles, particularly in paediatric populations. At the regulatory level, the approval pathways, quality control standards, and long-term safety monitoring systems for interventions such as FMT remain incomplete, with paediatric-specific frameworks being especially lacking. Establishing rigorous donor screening protocols, standardised manufacturing processes, and post-marketing surveillance tailored to children is essential to minimise risks. At the ethical level, it is crucial to fully inform parents and guardians about the limitations of the current evidence, the absence of long-term safety data in developing individuals, and the potential for unforeseen effects on growth, immune development, and the maturing gut–brain axis. Future research should prioritize several key directions. First, large-scale, multicenter, double-blind, randomized controlled trials with standardized sampling protocols are needed to validate therapeutic efficacy. Recent analysis of the SPARK-WGS cohort (7812 participants) demonstrates the feasibility and scientific value of well-powered microbiome studies in ASD (Manghi et al., 2024). Second, the integration of multi-omics technologies—including metagenomics, metaproteomics, and metabolomics—can elucidate complex host–microbe interactions (Zhong et al., 2026). Third, machine learning approaches offer promising diagnostic potential: recursive ensemble feature selection has identified reproducible microbiome signatures for ASD classification across diverse populations (Peralta-Marzal et al., 2024), while random forest models based on gut microbiota composition show high predictive accuracy, particularly in early childhood (D. Li et al., 2026). The integration of artificial intelligence with multi-omics data may enable precision microbiome-based interventions, but its application must be built upon a foundation of high-quality, longitudinal data.

## 6. Conclusions

This review systematically synthesizes current evidence linking gut microbiota to ASD, with a focus on microbial dysbiosis profiles, gut–brain axis mechanisms, and microbiota-targeted therapeutic strategies. Accumulating evidence indicates that individuals with ASD present with significant gut microbial dysbiosis, characterized by reduced beneficial bacteria, overgrowth of opportunistic pathogens, and fungal imbalances. These microbial alterations are thought to influence central nervous system development and function through multiple gut–brain axis pathways, including immune modulation, metabolic regulation, neural signaling, and neuroendocrine communication. Findings from animal studies and preliminary clinical evidence suggest that interventions targeting the gut microbiota—such as probiotics, prebiotics, and fecal microbiota transplantation—may hold promise for alleviating select ASD-related symptoms. Importantly, these therapeutic effects are likely mediated, at least in part, by microbial metabolites and their impact on gut–brain signaling. Nevertheless, existing studies in this field remain limited by several methodological shortcomings, and further rigorous investigation is needed to establish causal relationships and optimize clinical applications.

## Figures and Tables

**Table 1:** Gut microbiota alterations in ASD patients.

Bacterial Community Categories	Specific Changes	Functional Meaning	Reference
**Proportion of core phyla**			
*Firmicutes*/*Bacteroidetes* (*F*/*B*)	↑ or ↓	Associated with obesity and inflammatory states; a high *F*/*B* ratio may promote energy storage and a pro-inflammatory environment	(Stojanov et al., 2020; Alamoudi et al., 2022; Dan et al., 2020; X. Kong et al., 2019; Niu et al., 2019; Strati et al., 2017)
**Beneficial microbial flora ↓**			
*Prevotella*	↓	Carbohydrate fermentation and SCFA (acetic acid) production; influence of gut–brain signaling	(Kang et al., 2013; Kovatcheva-Datchary et al., 2015)
*Coprococcus*	↓	Butyrate production; maintenance of intestinal barrier integrity	(Kang et al., 2013; Kovatcheva-Datchary et al., 2015)
*Veillonellaceae*	↓	Lactic acid utilization and propionic acid production; regulate intestinal pH	(Kang et al., 2013; Kovatcheva-Datchary et al., 2015)
*Bifidobacterium* spp.	↓	Immune regulation; production of acetic/lactic acid; competitive exclusion of pathogens	(Gavzy et al., 2023; Tomova et al., 2015; L. Wang et al., 2011)
*Akkermansia muciniphila*	↓	Promote mucus layer renewal; maintain intestinal barrier; anti-inflammatory effect	(Gavzy et al., 2023; Tomova et al., 2015; L. Wang et al., 2011)
*Lactobacillus reuteri*	↓	Generate antimicrobial substances; modulate oxytocin signaling; improve social behavior	(Buffington et al., 2016).
*Lactobacillus johnsonii*	↓	Immune regulation; competitive inhibition of pathogenic bacteria	(Buffington et al., 2016).
*Bacteroides uniformis*	↓	Amino acid metabolism; regulation of neural excitation/inhibition balance	(Buffington et al., 2016).
*Bifidobacterium pseudolongum*	↓	Generate gamma-aminobutyric acid (GABA); neuroactive metabolites	(Buffington et al., 2016).
*Dialister*	↓	Its reduction is associated with impaired gut health	(Tao et al., 2025)
**Potential pathogenic bacteria ↑**			
*Escherichia* spp.	↑	Consumption of GABA; production of lipopolysaccharide (LPS); induction of neuroinflammation	(D. Wang et al., 2025)
*Desulfovibrio*	↑	Production of hydrogen sulfide (H_2_S); damage to intestinal epithelium; pro-inflammatory effects	(Morton et al., 2023; Socała et al., 2021; Zhou et al., 2024)
*Clostridium cluster XVIII*	↑	Some bacterial species produce neurotoxins; associated with neurodevelopmental abnormalities	(Morton et al., 2023; Socała et al., 2021; Zhou et al., 2024)
*Actinobacteria*	↑	Conditional pathogenic bacteria; Immune regulatory dysfunction	(Morton et al., 2023; Socała et al., 2021; Zhou et al., 2024)
*Sutterella*	↑ or ↓	Mucin degradation; associated with increased intestinal permeability	(Morton et al., 2023; Socała et al., 2021; Zhou et al., 2024)
*Betaproteobacteria*	↑	Conditional pathogenic bacteria; pro-inflammatory properties	(Morton et al., 2023; Socała et al., 2021; Zhou et al., 2024)
**Fungal community**			
*Candida albicans*	↑	Generate ammonia and toxins; induce abnormal behavior; synergize with bacterial dysbiosis	(Iovene et al., 2017)

**Table 2:** Methodological Comparison of 16S rRNA Sequencing and Metagenomic Sequencing in ASD Gut Microbiome Studies.

Feature	16S rRNA Sequencing	Metagenomic Sequencing (Shotgun)
Target	Amplifies variable regions (e.g., V3–V4) of 16S rRNA gene	Whole-genome DNA of all microbes
Taxonomic resolution	Typically genus level; species-level uncertain	Species and even strain-level resolution
Functional profiling	Not directly available (inferred by PICRUSt2, etc., with low accuracy)	Directly available (genes, pathways, KEGG/COG)
Detection of non-bacteria	Cannot detect fungi, viruses, archaea	Can detect all kingdoms (bacteria, fungi, viruses, archaea)
Cost	Low (~¥100–300/sample)	High (~¥600–1500/sample)
Data complexity	limited	High (requires more bioinformatics expertise)
Impact on ASD findings	Most early studies used 16S; identified consistent patterns (e.g., reduced *Bifidobacterium*), but unable to resolve species-level functional differences	Emerging studies using metagenomics reveal functional disruptions (e.g., SCFA synthesis pathways, tryptophan metabolism) not detectable by 16S

## Data Availability

Data sharing not applicable to this article as no datasets were generated or analyzed during the current study.
